# Early effects of antifibrotic therapy on respiratory muscle stiffness and dyspnea in SSc-ILD: a shear wave elastography study

**DOI:** 10.1007/s11739-026-04388-w

**Published:** 2026-05-18

**Authors:** Buğra Kerget, İsmail Çınar, Esen Saba Öktem, Aynur Daşdan, Sırma Merve Çetin, Mustafa Yeşilyurt, Alperen Aksakal, Fatih Alper

**Affiliations:** 1 Department of Pulmonary Diseases, Ataturk University School of Medicine, 25240 Yakutiye Erzurum, Turkey; 2 Department of Pulmonary Diseases, Aksaray University School of Medicine, 68200 Aksaaray, Turkey; 3 Department of Rheumatology, Erzurum City Hospital, 25240 Yakutiye Erzurum, Turkey; 4 Department of Rheumatology, Ataturk University School of Medicine, 25240 Yakutiye Erzurum, Turkey; 5 Department of Radiology, Ataturk University School of Medicine, 25240 Yakutiye Erzurum, Turkey

**Keywords:** Systemic sclerosis, Shear wave elastography, Anti-fibrotic treatment

## Abstract

Systemic sclerosis-associated interstitial lung disease (SSc-ILD) is characterized by microvascular injury and progressive fibrosis leading to respiratory impairment. This study aimed to evaluate the effect of anti-fibrotic therapy on intercostal and diaphragmatic shear wave elastography (SWE) measurements in patients with SSc-ILD and to assess their relationship with pulmonary function tests and Borg dyspnea scores. 30 patients with SSc-ILD receiving stable background therapy and 30 healthy controls were evaluated prospectively between January and August 2025. Intercostal and diaphragmatic SWE, pulmonary function tests, and Borg dyspnea scores were obtained before and three months after initiation of anti-fibrotic therapy. At three months, SSc-ILD patients showed significant improvement in FVC and FEV₁ (*p* = 0.009 and *p* = 0.02), while Borg scores and intercostal and diaphragmatic SWE values decreased (all *p* < 0.001). Elastography measurements regressed to levels comparable with controls. Changes in intercostal SWE correlated with ΔFVC and ΔFEV₁ (*R* = –0.769, *p* < 0.001 and *R* = –0.492, *p* = 0.007), and ΔBorg correlated with both intercostal and diaphragmatic SWE (*R* = 0.89 and *R* = 0.793, both *p* < 0.001). In multivariable analysis, elastographic improvement was the strongest determinant of dyspnea reduction (*p* < 0.001). Anti-fibrotic therapy initiation was associated with early reductions in respiratory muscle elastographic stiffness parameters and dyspnea in SSc-ILD. These short-term changes may reflect early biomechanical or functional alterations rather than definitive structural remodeling.

## Introduction

Systemic sclerosis (SSc) is a chronic autoimmune connective tissue disease characterized by microvascular injury and progressive fibrosis of the skin and internal organs [1]. Although its clinical course varies widely, lung involvement is both common and clinically significant. Interstitial lung disease and pulmonary vascular complications develop in a substantial proportion of patients and are the leading causes of SSc-related morbidity and mortality [2, 3]. These pulmonary manifestations highlight the need for careful monitoring and timely therapeutic intervention.

Recent international guidelines have emphasized the growing importance of anti-fibrotic therapy in systemic sclerosis-associated interstitial lung disease (SSc-ILD) [4]. Evidence from randomized clinical trials has demonstrated that agents such as nintedanib can slow the rate of lung function decline, particularly in patients with progressive fibrotic involvement [5]. Accordingly, both the ATS/ERS and EULAR recommendations now support the use of anti-fibrotic treatment in selected SSc-ILD patients, either as monotherapy or in combination with immunomodulatory agents [6–8]. Despite these advances, treatment response remains heterogeneous, and current decision-making still relies heavily on serial pulmonary function testing, which may not fully capture early or extra-pulmonary changes [6].

Despite the increasing use of anti-fibrotic agents in SSc-associated interstitial lung disease, their impact on patient-reported dyspnea remains less clear. While major clinical trials have demonstrated that therapies such as nintedanib can slow the decline in pulmonary function, these benefits have not consistently translated into symptomatic improvement [9, 10]. Moreover, anti-fibrotic drugs are known to modulate fibrogenic pathways beyond the lung, and experimental models have shown reductions in skeletal muscle fibrosis, suggesting a broader biological effect on tissue stiffness [11, 12]. However, whether such mechanisms influence respiratory muscle properties or contribute to dyspnea relief in SSc-ILD has not been established [13, 14]. Traditional monitoring tools, such as spirometry and diffusing capacity, do not fully capture these potential extra-pulmonary changes, underscoring the need for complementary, noninvasive modalities that can better reflect the structural determinants of breathlessness [15–17].

In this study, we aimed to investigate the relationship between anti-fibrotic treatment-related changes in intercostal and diaphragmatic SWE values and dyspnea improvement in patients with SSc-ILD, and to determine whether SWE can serve as a complementary monitoring tool alongside pulmonary function tests.

## Methods

### Study design

This study was designed as a prospective, longitudinal, and observational clinical investigation. Patients with systemic sclerosis who developed SSc-ILD during routine rheumatology follow-up and were referred to our tertiary center between January 2025 and August 2025 were consecutively enrolled. All participants underwent baseline clinical evaluation, pulmonary function testing (PFT), Borg dyspnea scoring, and intercostal and diaphragmatic SWE prior to initiation of anti-fibrotic therapy. The same assessments were repeated after three months of treatment to evaluate temporal changes. No randomization or additional intervention beyond standard clinical management was performed. The study was conducted in accordance with the principles of the Declaration of Helsinki, and written informed consent was obtained from all participants before enrollment.

### Study population

Patients were eligible for inclusion if they had a confirmed diagnosis of SSc-ILD, were on stable background therapy, were able to complete follow-up assessments, and demonstrated adequate cooperation for spirometry and elastography measurements. Exclusion criteria were: prior use of anti-fibrotic therapy within the last six months or changes in background immunosuppressive treatment during the same period; conditions that could affect elastography measurements (including neuromuscular diseases, myositis, hypothyroidism, diabetes with peripheral neuropathy, chronic kidney disease, or systemic muscle-affecting medications such as statins or high-dose corticosteroids); active pulmonary infection; connective tissue disease-associated pulmonary hypertension; significant cardiac dysfunction; pregnancy; malignancy; and inability to perform pulmonary function tests. Additionally, patients who did not attend the three-month follow-up visit or had incomplete data were excluded.

**Fig. 1:**
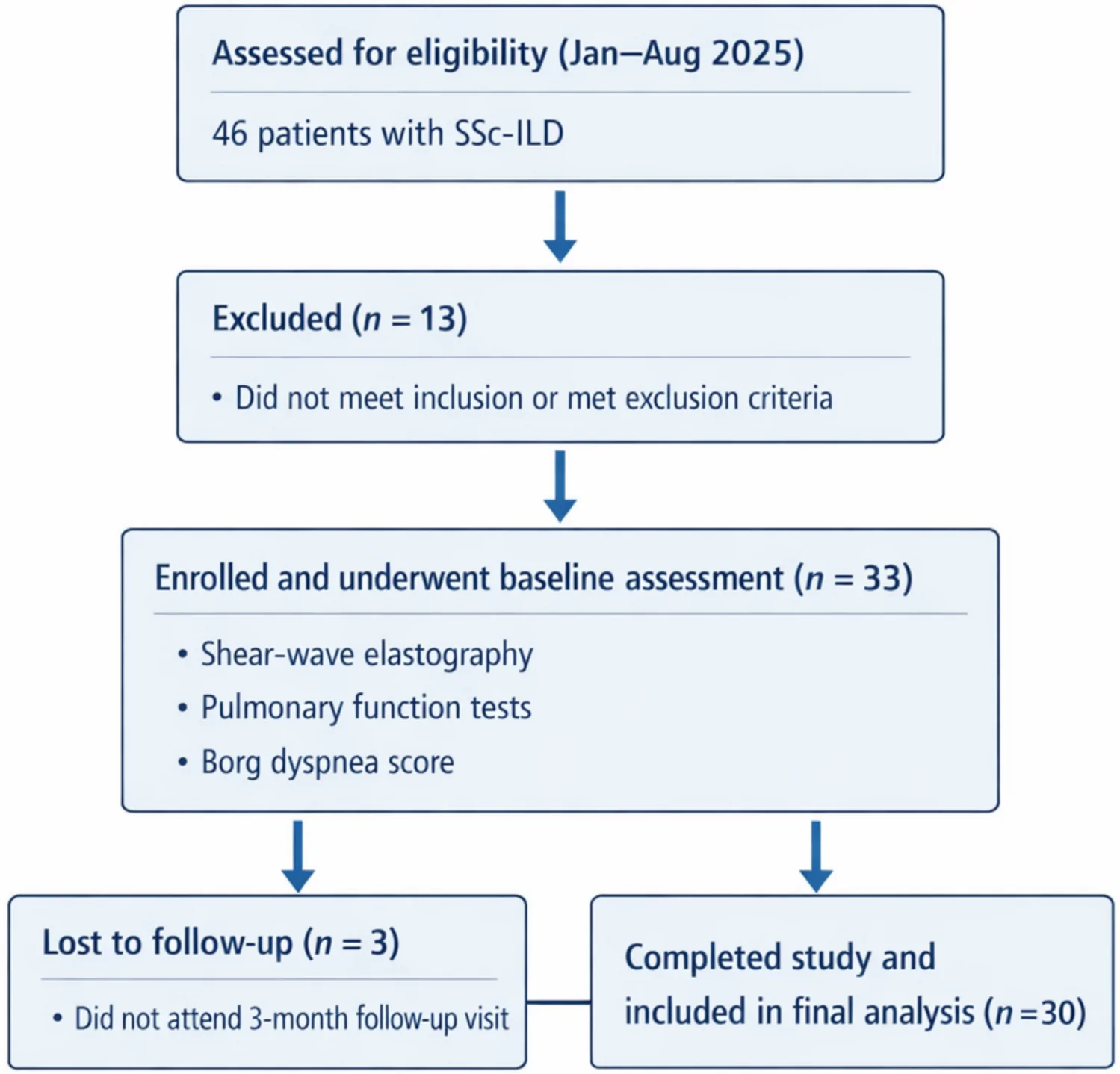
Flowchart of patient enrollment, exclusions, follow-up, and final analysis

During the study period, 46 patients with SSc-ILD were screened for eligibility. Thirteen patients were excluded according to the predefined exclusion criteria. Of the remaining 33 patients who underwent baseline assessments, three did not attend the three-month follow-up visit and were excluded from the final analysis. Thus, a total of 30 patients completed the study (Fig. 1).

All patients fulfilled the 2023 ACR/EULAR classification criteria for SSc and had been receiving standard anti-inflammatory therapy according to current rheumatology guidelines before the diagnosis of SSc-ILD [18]. After diagnosis, patients received background therapy individualized according to routine rheumatology practice. This included hydroxychloroquine (5 mg/kg/day) and nifedipine (60 mg/day) for Raynaud’s phenomenon. Mycophenolate mofetil was prescribed in selected patients based on overall disease assessment, including pulmonary involvement and clinician judgment, rather than as a standardized treatment for cutaneous manifestations. Patients were followed monthly with assessment of Raynaud’s symptom control, screening for connective tissue disease-associated pulmonary hypertension, and evaluation of cutaneous involvement using the modified Rodnan skin score (mRSS).

In addition, some patients were receiving supportive treatments according to individual clinical needs, including proton pump inhibitors for gastroesophageal reflux symptoms, antihypertensive agents, calcium/vitamin D supplementation, and low-dose antiplatelet therapy when indicated. No additional immunosuppressive or disease-modifying therapies were initiated during the study period, and no relevant changes in background treatment occurred during follow-up.

High-resolution computed tomography (HRCT) was performed in all patients at baseline as part of routine clinical evaluation. Fibrotic involvement was defined by the presence of reticulation with traction bronchiectasis and/or honeycombing, with or without ground-glass opacities. The predominant radiological pattern was classified as fibrotic nonspecific interstitial pneumonia (fNSIP) or usual interstitial pneumonia (UIP)-like pattern according to current international criteria.

Anti-fibrotic therapy was initiated in accordance with contemporary clinical practice guidelines for systemic sclerosis-associated interstitial lung disease and progressive pulmonary fibrosis. Specifically, anti-fibrotics were considered for patients with fibrotic ILD on high-resolution computed tomography who fulfilled the ATS/ERS/JRS/ALAT 2022 criteria for progressive pulmonary fibrosis, defined by the presence of at least two of the following within the previous year with no alternative explanation: worsening respiratory symptoms; physiological disease progression (absolute decline in forced vital capacity ≥ 5% predicted or absolute decline in DLCO ≥ 10% predicted); and/or radiological evidence of disease progression. Anti-fibrotic therapy was initiated after optimization of background immunomodulatory treatment [7]. Only clinically stable patients with confirmed SSc-ILD were subsequently referred to our center for initiation of anti-fibrotic therapy, and nintedanib 300 mg/day (Ofev®) was started as standard treatment. Baseline clinical assessment, pulmonary function testing, Borg dyspnea scoring, and intercostal and diaphragmatic elastography were performed before antifibrotic initiation, with the same evaluations repeated after three months. An age-and-sex-matched control group of 30 healthy volunteers without known cardiopulmonary disease was also included to provide reference elastography values rather than to evaluate therapeutic efficacy.

Furthermore, no changes in background immunosuppressive therapy, including dose adjustments or initiation of new agents, occurred during the three-month follow-up period. None of the patients received systemic corticosteroid dose escalation or tapering during the study period. Therefore, all observed changes in pulmonary function, elastography measurements, and dyspnea scores were evaluated under stable background treatment conditions. During the three-month follow-up period, nintedanib was generally well tolerated. A temporary dose reduction was required in a small number of patients due to gastrointestinal adverse effects; however, no patients required permanent discontinuation of anti-fibrotic therapy. All patients were able to complete the planned follow-up assessments.

Connective tissue disease-associated pulmonary hypertension was systematically excluded in all patients prior to study enrollment. All participants underwent comprehensive transthoracic echocardiographic evaluation within three months before inclusion. Estimated systolic pulmonary artery pressure (sPAP) was calculated using tricuspid regurgitation jet velocity, and right ventricular size and function were assessed according to standard echocardiographic criteria. Patients with an estimated sPAP > 35 mmHg, right ventricular enlargement or dysfunction, or other echocardiographic findings suggestive of pulmonary hypertension were excluded from the study. None of the included patients had clinical or echocardiographic indications warranting right heart catheterization at the time of enrollment.

### Pulmonary function testing

Pulmonary function testing was performed in accordance with the ATS/ERS 2019 recommendations [19]. Patients were informed of all pre-test requirements, including withholding short-acting bronchodilators and avoiding heavy physical activity prior to testing. The maneuver was demonstrated by the technician, and each participant performed at least three acceptable and reproducible spirograms. Only measurements meeting ATS/ERS acceptability and repeatability criteria were included in the analysis. Spirometry and diffusing capacity were assessed using a Jaeger MasterScreen PFT Pro system (CareFusion, Höchberg, Germany), which automatically calculated reference values and lower limits of normal based on population-adjusted prediction equations. All assessments were performed by the same technician at baseline and at the three-month follow-up.

### Shear wave elastography measurements

All elastography examinations were performed using a high-resolution ultrasonography system (EpiqElite, Philips Medical Systems) equipped with a 22–2 MHz transducer. Measurements were obtained at baseline and at the three-month follow-up under standardized conditions. For diaphragmatic assessment, participants were placed in the left lateral decubitus position. The probe was positioned beneath the rib cage between the anterior axillary line and the 6th–12th intercostal spaces without applying compressive force. Gain and depth settings were optimized, and three 1-mm circular regions of interest (ROIs) were placed within the elastography window; the mean shear modulus was recorded in kilopascals (kPa).

**Fig. 2:**
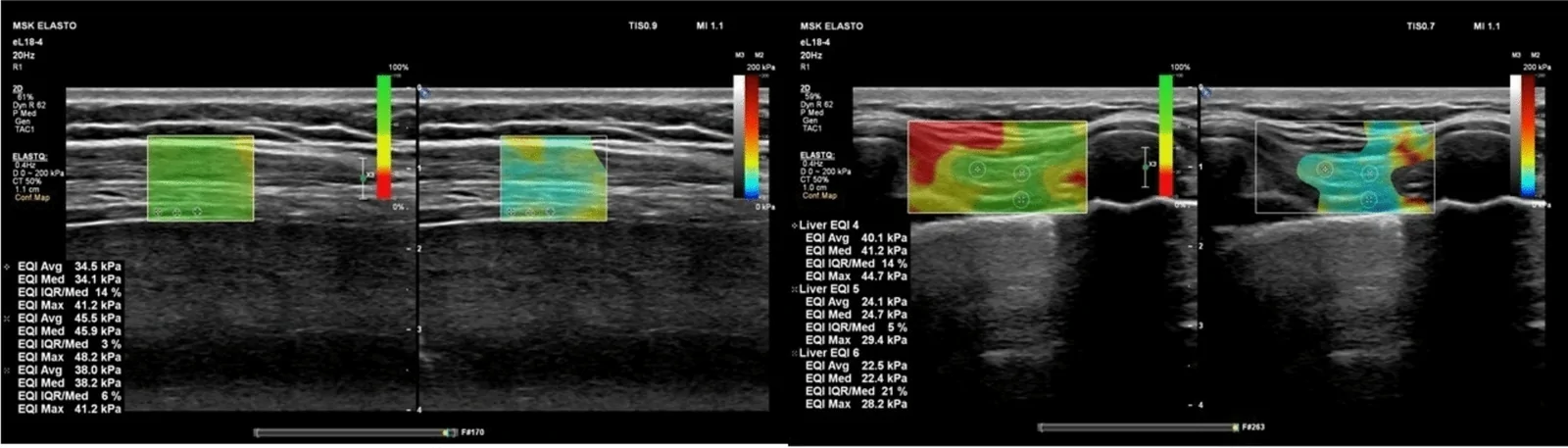
2D-SWE measurement of the elastic modulus of the diaphragm and intercostal muscles

Intercostal measurements were obtained in the supine position by placing the transducer over the anterior chest wall. Images were acquired while the participant maintained end-inspiratory breath-hold to minimize motion artifact. A 3-mm circular ROI was used, and values were averaged after three consecutive measurements. An IQR/MED ratio of < 30% was required to ensure image quality and measurement reliability (Fig. 2). All shear-wave elastography examinations were performed by a single radiologist with more than 13 years of experience in thoracic and musculoskeletal ultrasonography. The operator was aware that the participants had systemic sclerosis-associated interstitial lung disease as complete diagnostic blinding was not feasible in this prospective clinical setting, but was blinded to pulmonary function test results, Borg dyspnea scores, treatment stage (baseline vs. follow-up), and other clinical data at the time of image acquisition and analysis.

### Dyspnea assessment

Dyspnea severity was evaluated using the modified Borg scale, ranging from 0 (no breathlessness) to 10 (maximal breathlessness). All assessments were performed at baseline and at the three-month follow-up under standardized conditions. Patients were instructed on the use of the scale prior to scoring to ensure consistent understanding. Measurements were obtained at rest, immediately before pulmonary function testing, with the patient seated and breathing room air. No verbal or visual cues were provided during scoring to avoid influencing responses. The same clinician administered the scale at both visits to minimize inter-observer variability.

### Outcome measures

The primary outcome of the study was the change in dyspnea severity over the three-month treatment period, assessed using the modified Borg scale. The primary analytical objective was to determine the association between Borg score variation and changes in intercostal and diaphragmatic SWE measurements.

Secondary outcomes included:

- changes in pulmonary function parameters (FVC, FEV₁, and FEV₁/FVC ratio) between baseline and the three-month follow-up,
- comparison of elastography measurements with matched healthy controls at baseline,
- The relationship between variations in elastography values and pulmonary function test results,
- The proportion of patients demonstrating clinically meaningful improvement in dyspnea (defined as a ≥ 1-point reduction in Borg score).

All outcomes were evaluated using paired measurements obtained before initiation of antifibrotic therapy and at the three-month follow-up.

### Statistical analysis

Statistical analysis was performed using IBM SPSS Statistics, version 27.0 (IBM Corp., Armonk, NY, USA). The distribution of continuous variables was assessed using the Shapiro–Wilk test. Descriptive data were expressed as mean ± standard deviation for normally distributed variables and as median (minimum–maximum) for non-normally distributed variables. Baseline comparisons between the patient and control groups were performed using the independent samples t-test or the Mann–Whitney U test, as appropriate. Changes in pulmonary function parameters, Borg dyspnea scores, and elastography measurements between baseline and the three-month follow-up were analyzed using the paired t-test or the Wilcoxon signed-rank test. Categorical variables were compared using the chi-square test or Fisher’s exact test when required. Correlation analysis between changes in elastography measurements and variations in pulmonary function or Borg scores were evaluated using Pearson’s correlation coefficient. For these analyses, ΔFVC and ΔFEV₁ were calculated as the difference between the third-month and baseline values (third month − baseline), whereas Δ intercostal SWE, Δ diaphragmatic SWE, and Δ Borg were calculated as the difference between baseline and third-month values (baseline − third month). A multivariable linear regression model was constructed to identify independent predictors of dyspnea improvement, including age, Δ intercostal SWE, and Δ diaphragmatic SWE. Model assumptions were checked prior to interpretation. A two-tailed *p* value of < 0.05 was considered statistically significant.

## Results

The average age of the patients in the study was 58.3 ± 7.9 years, while the average age of the healthy control group was 60.4 ± 10.4 years. Statistical comparison between the patient and control groups showed no significant difference (*p* = 0.34). 28 patients (93%) in the study were female, and 28 controls (93%) were also female. 16 patients (53.3%) had hypertension, and two (6.7%) had diabetes mellitus. In the control group, ten patients (33.3%) had hypertension.

A comparison of pulmonary function test parameters, Borg dyspnea scale, intercostal, and diaphragmatic SWE levels in SSc-ILD patients before and at the third month of antifibrotic treatment is shown in Table 1. Accordingly, it was observed that FVC (Lt), FEV1 (%), and FVC (%) levels increased compared to baseline levels at the third month of antifibrotic treatment (*p* = 0.01, 0.02, 0.009, respectively). A statistically significant decrease was observed in the Borg dyspnea scale, intercostal, and diaphragmatic SWE levels at the third month of treatment (*p* < 0.001 for all). In the comparison of pre-treatment intercostal and diaphragmatic SWE levels with the control group, it was observed that the elastography levels, which were higher in SSc-ILD patients (*p* < 0.001 for both), decreased to levels similar to those of the control group in the third month of treatment (*p* = 0.9, 0.08 respectively).

**Table 1:** Comparison of respiratory function test parameters, BORG dyspnea scale, intercostal and diaphragmatic SWE levels in SSc-ILD patients before starting antifibrotic treatment and at the third month of treatment

	Before anti-fibrotic treatment (*n* = 30) Median (Min -Max)	Third month of antifibrotic treatment (*n* = 30) Median (Min -Max)	*p*			
FEV1/FVC (%)	78.5 (52.6–96.7)	78.7 (52.6–96.7)	0.79			
FEV1 (Lt)	2.11 (1.35–3.74)	2.15 (1.36–3.74)	0.06			
FVC (Lt)	2.8 (1.58–5.03)	2.86 (1.63–5.11)	0.01			
FEV1 (%)	89 (65–141)	101 (77–141)	0.02			
FVC (%)	96 (72–130)	100 (72–130)	0.009			
PEF25-75 (%)	84 (35–106)	82 (28–111)	0.36			
DLCO (%)	69 (33–121)	74 (34–121)	0.33			
DLCO/Va (Kco) (%)	86 (46–130)	83 (44–148)	0.76			
Borg dyspnea scale	5 (1–7)	4 (0–6)	< 0.001			
	Before anti-fibrotic treatment (*n* = 30) Median (Min -Max)	Third month of antifibrotic treatment (*n* = 30) Median (Min -Max)		Control Group (*n* = 30) Median (Min -Max)	p*	p**
İntercostal SWE (kPa)	31.4 (12–65.3)	25.6 (12–36.8)	< 0.001	25 (16.2–30.3)	< 0.001	0.9
Diaphragmatic SWE (kPa)	45.9 (26.9 − 81)	34 (17.6–63.4)	< 0.001	37 (28–46.9)	< 0.001	0.08

p*: Comparison of pre-treatment values with the control group, p**: Comparison of post-treatment values with the control group, *FVC* Forced vital capacity, *FEV1* Forced expiratory volume in 1 s, *DLCO* Diffusing capacity of the lung for carbon monoxide, *DLCO/VA* Ratio of DLCO to alveolar volume

Multivariable linear regression analysis including age, Δ intercostal SWE, and Δ diaphragmatic SWE as predictors of change in the Borg dyspnea score is presented in Table 2. Decreases in both intercostal and diaphragmatic SWE values were significantly associated with improvement in the Borg dyspnea score (both *p* < 0.001).

**Table 2:** Multivariable linear regression analysis of factors affecting changes in Borg dyspnea score

Variable	B	Standard Error	Standardized Beta	t	*p* value	95% CI for B
Constant	− 0.576	0.275	–	− 2.093	0.046	− 1.142 to − 0.010
Age (year)	0.009	0.005	0.134	2.098	0.046	0.000 to 0.019
Δ Intercostal SWE (kPa)	0.040	0.005	0.615	8.195	< 0.001	0.030 to 0.050
Δ Diaphragmatic SWE (kPa)	0.050	0.010	0.376	5.056	< 0.001	0.030 to 0.071

**Fig. 3:**
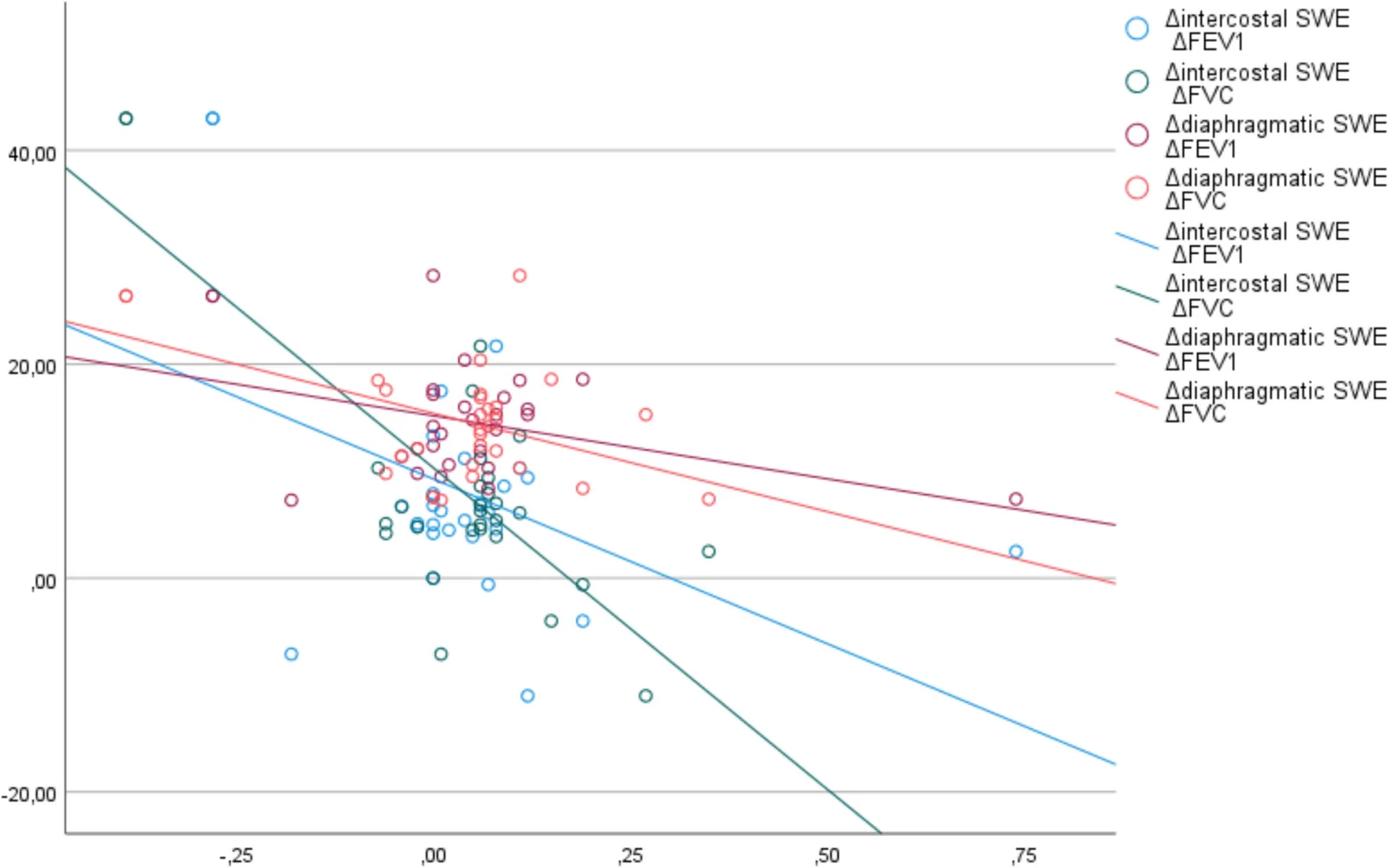
Correlation analysis of changes in intercostal and diaphragmatic SWE levels with changes in FEV1 and FVC

Correlation analysis of Δ intercostal, Δ diaphragmatic SWE, ΔFEV1, and ΔFVC levels is presented in Fig. 3. Accordingly, a statistically significant negative correlation was found between Δ intercostal SWE and both ΔFVC and ΔFEV1 (*R* = −0.769, *p* < 0.001; *R* = −0.492, *p* = 0.007, respectively). Additionally, a statistically significant negative correlation was observed between Δ diaphragmatic SWE and ΔFVC (*R* = −0.492, *p* = 0.007).

**Fig. 4:**
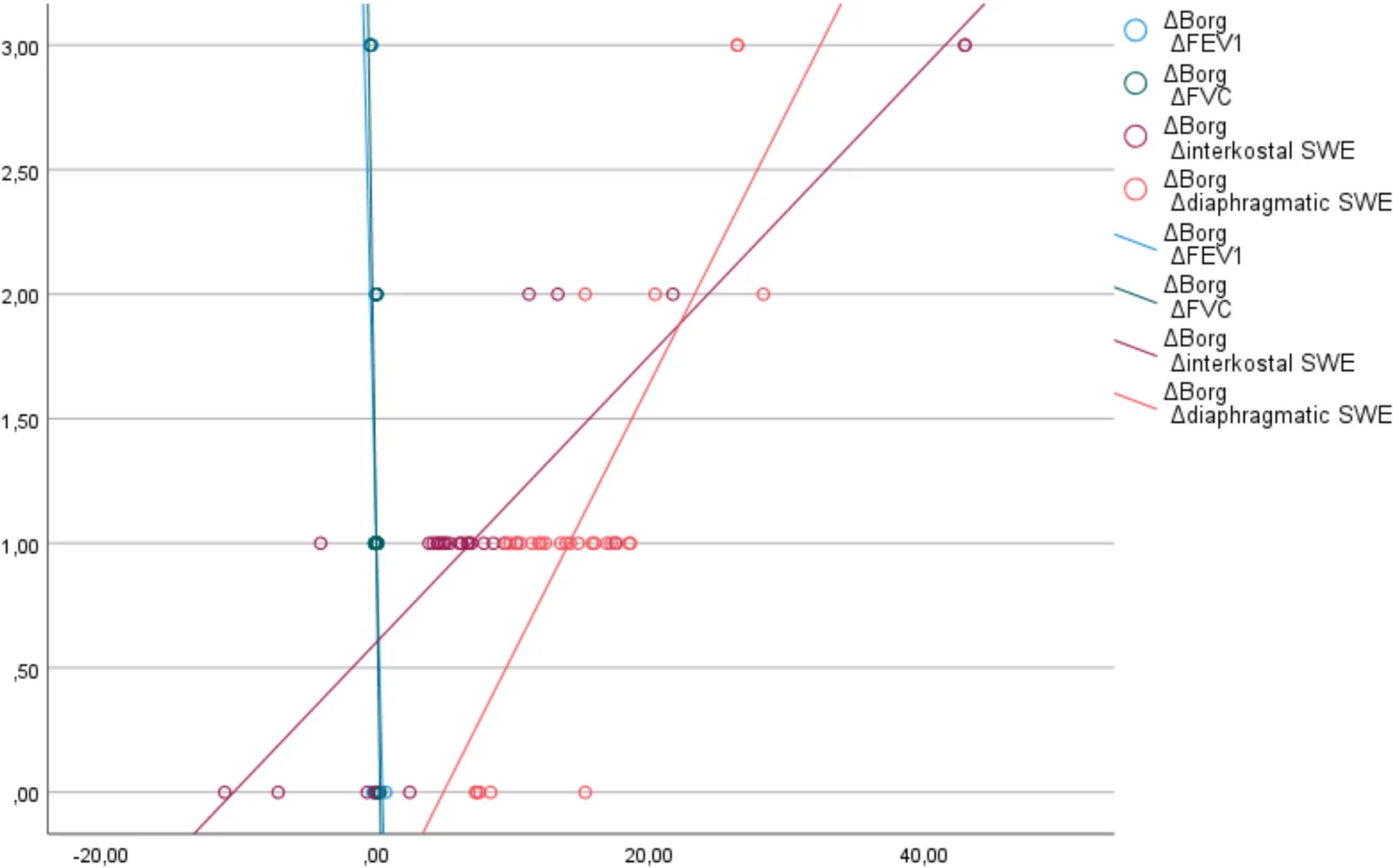
Correlation analysis of the changes in the Borg dyspnea scale and respiratory function test parameters, as well as intercostal and diaphragmatic SWE levels

Correlation analysis of changes in the Borg dyspnea scale and pulmonary function test parameters, as well as intercostal and diaphragmatic SWE levels, is presented in Fig. 4. Accordingly, a positive, statistically significant difference was observed between ΔBorg and Δintercostal and Δdiaphragmatic SWE levels, while a negative, statistically significant difference was noted between ΔFVC and ΔFEV1 (*R* = 0.89, *p* < 0.001; *R* = 0.793, *p* < 0.001; *R* = −0.711, *p* < 0.001; *R* = −0.507, *p* < 0.001, respectively).

## Discussion

To the best of our knowledge, this is the first prospective study to assess early changes in intercostal and diaphragmatic shear-wave elastography in patients with systemic sclerosis-associated interstitial lung disease receiving antifibrotic therapy, and to examine their relationship with dyspnea. Previous studies in SSc-ILD have largely focused on pulmonary function, imaging progression, or patient-reported outcomes, whereas data on respiratory muscle biomechanics are limited. By integrating elastographic assessment with pulmonary function testing and dyspnea evaluation, this study provides novel insight into extrapulmonary mechanical contributors to symptom burden in SSc-ILD.

In this study, intercostal and diaphragmatic elastography measurements obtained at baseline, before initiation of antifibrotic therapy, were significantly higher in patients compared with healthy controls, suggesting that structural stiffness of the respiratory muscles may be affected even at the time of SSc-ILD diagnosis. After initiation of antifibrotic therapy, improvements were observed over the three-month follow-up in both pulmonary function parameters and dyspnea scores; in parallel, elastography values markedly decreased and regressed to levels comparable with those of the control group. In the multivariable regression analysis, improvement in elastography measurements emerged as an independent determinant of dyspnea reduction, indicating that respiratory muscle mechanics may directly contribute to symptomatic relief. Furthermore, three-month changes in elastography demonstrated a significant positive correlation with increases in FEV₁ and FVC, and an expected inverse correlation with reductions in Borg scores, supporting the association between muscle stiffness, functional capacity, and clinical symptom burden.

Pulmonary involvement is highly prevalent in systemic sclerosis, with ILD detected in approximately 35–50% of patients at the time of diagnosis and increasing in frequency during the disease course [20]. Because ILD may remain clinically silent in the early stages, irreversible fibrotic progression can occur before functional impairment becomes evident. For this reason, recent ATS/ERS and EULAR recommendations emphasize early evaluation and timely initiation of antifibrotic therapy not only in patients with established ILD but also in those at risk of progression [7, 8, 18, 21]. This approach reflects the growing shift toward preventing long-term decline by intervening as close to diagnosis as possible [22].

Extrapulmonary involvement in systemic sclerosis extends beyond the lung and frequently includes skeletal muscle abnormalities characterized by fibrosis, reduced capillary density, and inflammatory or non-inflammatory myopathy [23, 24]. These changes may contribute to impaired respiratory mechanics through decreased chest wall compliance and reduced strength of accessory muscles, potentially amplifying breathlessness independent of lung parenchymal involvement [25]. While antifibrotic therapies have demonstrated a consistent ability to slow pulmonary function decline in systemic sclerosis-associated interstitial lung disease, improvements in dyspnea have been modest and inconsistent across clinical trials, suggesting that factors outside the lung may influence symptom perception [9, 26]. Experimental studies have shown that antifibrotic agents can modulate profibrotic signaling pathways and reduce collagen accumulation within skeletal muscle tissue, raising the possibility of broader biological effects beyond the lung [11, 27, 28]. Although a direct biological effect of antifibrotic therapy on respiratory muscle tissue is biologically plausible, the present study was not designed to establish a mechanism. The observed reductions in SWE values may instead reflect multiple interacting processes, including altered tissue compliance, inflammatory activity, chest wall mechanics, or changes in respiratory muscle loading conditions. However, whether such mechanisms translate into clinically meaningful changes in respiratory muscle properties remains unknown, underscoring the need for complementary assessment tools capable of capturing both pulmonary and extrapulmonary contributors to dyspnea.

In our study, consistent with the literature, the majority of cases were female. In addition to anti-inflammatory treatment at diagnosis, antifibrotic treatment was started according to current guideline recommendations, and notable improvements in FVC and FEV₁ were observed at three-month follow-up. Although the observed improvements in pulmonary function were modest and no significant change in DLCO was detected, these findings may still represent early treatment-associated changes during a short follow-up period. While intercostal and diaphragmatic elastography measurements were initially higher than those of healthy controls, these values decreased to levels similar to the control group by the end of follow-up, suggesting early changes in respiratory muscle mechanical properties. Although no statistically significant change was observed in diffusion capacity, the notable improvement in dyspnea scores indicates that symptoms cannot be explained solely by gas exchange. While the intercostal muscles are vital for chest wall expansion and forced breathing, the diaphragm tends to be affected later in life due to its primary role in resting breathing. This physiological difference aligns with the stronger correlation between changes in intercostal elastography and FEV₁ and FVC in our study. Taken together, these findings suggest that antifibrotic therapy appears not only to help preserve lung function but also to be associated with favorable changes in respiratory muscle mechanical properties. The reduction in dyspnea levels with treatment indicates that respiratory comfort goes beyond changes in lung function. A one-point reduction in Borg score is commonly considered a clinically meaningful change in perceived dyspnea, which supports the potential symptomatic relevance of the observed findings. In fact, the change in dyspnea shows a positive correlation with increases in FVC and FEV₁ and, as expected, with significant decreases in intercostal and diaphragmatic elastography values. In multivariate regression analysis, elastographic change emerged as a stronger predictor of dyspnea improvement than other parameters, indicating that respiratory relaxation in patients may be more closely linked to reduced muscle stiffness. These findings support the idea that dyspnea in SSc-ILD may be affected not only by parenchymal involvement but also by alterations in chest wall and respiratory muscle mechanics.

The relatively short three-month follow-up period should be interpreted cautiously. Established fibrosis is unlikely to undergo substantial reversal within such a limited timeframe. Therefore, the reductions observed in intercostal and diaphragmatic SWE values may represent early changes in tissue mechanical behavior, inflammation-related stiffness, muscle loading conditions, or compliance rather than confirmed regression of fibrosis. Nevertheless, detection of such early changes may still be clinically relevant, particularly if associated with symptomatic improvement.

This study has several limitations. The sample size was small and derived from a single center, which may restrict external generalizability and may have led to overestimation of some observed associations; however, systemic sclerosis is a rare disease and strict inclusion criteria were necessary to obtain a homogeneous, treatment-naïve cohort. The follow-up period was limited to three months, allowing assessment of early treatment-associated changes but not definitive structural remodeling or long-term outcomes. Moreover, the absence of a parallel comparator group of SSc-ILD patients not receiving antifibrotic therapy limits causal interpretation of the observed longitudinal changes. Residual confounding related to heterogeneous background therapies, baseline disease severity, and selection of clinically stable patients also cannot be excluded. Furthermore, elastography findings could not be validated with biopsy or advanced imaging, formal reproducibility metrics were not assessed, and residual operator-dependent bias cannot be excluded. Despite these constraints, standardized measurements and consistent imaging by a single experienced operator strengthen the reliability of the results.

In conclusion, this study suggests that patients with systemic sclerosis may exhibit increased intercostal and diaphragmatic elastographic stiffness parameters at diagnosis, with early reductions observed after initiation of antifibrotic therapy during the first three months of follow-up. The parallel decrease in elastography values and dyspnea severity, together with their association with changes in pulmonary function, suggests that respiratory muscle mechanics may contribute to symptomatic improvement beyond pulmonary function alone. Although these findings support the potential role of elastography as a complementary monitoring tool during the early treatment period, larger studies with longer follow-up are needed to determine the durability, structural significance, and clinical relevance of these observations.
